# Mesenchymal Stem Cells in a Transgenic Mouse Model of Multiple System Atrophy: Immunomodulation and Neuroprotection

**DOI:** 10.1371/journal.pone.0019808

**Published:** 2011-05-18

**Authors:** Sylvia Stemberger, Angelika Jamnig, Nadia Stefanova, Günter Lepperdinger, Markus Reindl, Gregor K. Wenning

**Affiliations:** 1 Divison of Clinical Neurobiology, Department of Neurology, Innsbruck Medical University, Innsbruck, Austria; 2 Clinical Department of Neurology, Innsbruck Medical University, Innsbruck, Austria; 3 Extracellular Matrix Research, Institute for Biomedical Aging Research, Austrian Academy of Science, Innsbruck, Austria; Charité Universitätsmedizin Berlin, NeuroCure Clinical Research Center, Germany

## Abstract

**Background:**

Mesenchymal stem cells (MSC) are currently strong candidates for cell-based therapies. They are well known for their differentiation potential and immunoregulatory properties and have been proven to be potentially effective in the treatment of a large variety of diseases, including neurodegenerative disorders. Currently there is no treatment that provides consistent long-term benefits for patients with multiple system atrophy (MSA), a fatal late onset α-synucleinopathy. Principally neuroprotective or regenerative strategies, including cell-based therapies, represent a powerful approach for treating MSA. In this study we investigated the efficacy of intravenously applied MSCs in terms of behavioural improvement, neuroprotection and modulation of neuroinflammation in the (PLP)-αsynuclein (αSYN) MSA model.

**Methodology/Principal Findings:**

MSCs were intravenously applied in aged (PLP)-αSYN transgenic mice. Behavioural analyses, defining fine motor coordination and balance capabilities as well as stride length analysis, were performed to measure behavioural outcome. Neuroprotection was assessed by quantifying TH neurons in the substantia nigra pars compacta (SNc). MSC treatment on neuroinflammation was analysed by cytokine measurements (IL-1α, IL-2, IL-4, IL-5, IL-6, IL-10, IL-17, GM-CSF, INFγ, MCP-1, TGF-β1, TNF-α) in brain lysates together with immunohistochemistry for T-cells and microglia.

Four weeks post MSC treatment we observed neuroprotection in the SNc, as well as downregulation of cytokines involved in neuroinflammation. However, there was no behavioural improvement after MSC application.

**Conclusions/Significance:**

To our knowledge this is the first experimental approach of MSC treatment in a transgenic MSA mouse model. Our data suggest that intravenously infused MSCs have a potent effect on immunomodulation and neuroprotection. Our data warrant further studies to elucidate the efficacy of systemically administered MSCs in transgenic MSA models.

## Introduction

Multiple system atrophy (MSA) denotes an adult-onset neurodegenerative disorder of relentless progression and unknown aetiology that is clinically characterized by the variable combination of autonomic failure, levodopa-unresponsive parkinsonism, cerebellar ataxia, and pyramidal signs. MSA affects men and woman equally, usually starting in the sixth decade and progresses rapidly with death occurring after an average of nine years [1]. Pathological features cover selective neuronal cell loss and gliosis in the basal ganglia, cerebellum, pontine and inferior olivary nuclei, pyramidal tract, intermediolateral cell column and Onuf's nucleus [2].

Morphologically, MSA is considered a primary oligodendrogliopathy based on the cellular hallmark, the glial cytoplasmic inclusions (GCIs) [3]. GCIs contain primarily α-synuclein (αSYN) and hence link MSA with other α-synucleinopathies, such as Parkinson's disease (PD) and dementia with Lewy Bodies (DLB) [4], [5]. Still the underlying mechanism of αSYN aggregates, which appear to play a fundamental role in disease pathogenesis, remains to be determined. However, several molecular and cellular changes, including oxidative stress, mitochondrial dysfunction and apoptotic processes might be involved in neuronal degeneration [1], [6], [7].

Microglial activation has been reported to parallel the neuronal multisystem degeneration in MSA [8], suggesting neuroinflammation as a key pathogenic mechanism comparable to findings in PD [9]. During the last years, studies analysing polymorphism of genes involved in inflammatory processes, such as interleukin-1alpha (IL-α), interleukin-1beta (IL-1β), interleukin-8, intercellular adhesion molecule-1 and tumor necrosis factor showed elevated MSA risk [10]. These studies point towards a possible role of neuroinflammation in MSA pathogenesis.

At present, MSA therapy is only symptomatic and mainly targets parkinsonism and autonomic failure [2] as there is no drug treatment that provides MSA patients with consistent long-term benefits. Neuroprotective or regenerative strategies, including neurotransplantation, appear to be an alternative therapeutic approach for managing MSA patients. Experimentally, different cell types for neural restoration in MSA have been tried. E13 whole ganglionic eminence grafts survive and exert functional benefit in toxin-based MSA models [11]–[13]. Moreover, survival, integration and functional benefit of E13 ventral mesencephalic (VM) grafts in toxin-based MSA models has been demonstrated [14] .

A particular type of stem cells which is considered clinically more attractive, ethically less problematic and exhibiting immunological properties that make them superior over other cell types are mesenchymal stem cells (MSCs). First described by Friedenstein and colleagues [15], as a population of bone marrow (BM) cells, also known as fibroblast-colony-forming cells, which adhere to cell culture plastic surfaces, these cells were shown to differentiate into many mesodermal derivatives, such as adipocytes, osteocytes and chondrocytes, *in vitro* and *in vivo* when exposed to appropriate stimuli [16], [17].

The MSCs' ability to differentiate into neural-like and glial-like cells could be shown, albeit *in vitro* only [18], [19]. Based on this and similar results, subsequent studies have been initiated and designed in order to prove these cells' potential to support neuroregeneration and also to provoke their immunomodulatory properties in regions, which are actually void of MSC (reviewed in [20]). This particular body of literature is vastly growing, yet lacking strong *in vivo* evidence which demonstrates that MSCs, unless they are ectopically placed there or infused in large numbers, are indeed capable of bringing forth neuro-ectodermal derivatives. In light of this, many findings and interpretations remain elusive [21].

Long-term clinical and radiological effects of MSCs in patients with MSA have been described by Lee and co-workers in 2008 [22]. In an open-label study design, the neurological deficits in 11 patients with the cerebellar type of MSA (MSA-C), who received consecutively intra-arterial and three repeated intravenous injections for three months, were compared with non-treated MSA patients, demonstrating a delay in progression of neurological deficits after MSC therapy [22]. A recent study by the same group investigated successful neuroprotective and immunomodulatory effects of human MSCs in a double-toxin induced animal model of MSA-P [23]. However this double-toxin induced model solely represents striatonigral-like pathology, without reproducing oligodendroglial inclusion pathology, mediating secondary neuronal multisystem degeneration [3].

At present to our knowledge, there is no experimental evidence for the neuroregenerative potential of MSCs in transgenic mice, overexpressing oligodendroglial αSYN, mimicking important aspects of MSA, such as neuronal loss linked to MSA-like progressive autonomic failure, cerebellar ataxia and parkinsonism, GCI pathology, astrogliosis and microglial activation. For this reason we applied murine MSCs intravenously in aged (PLP)-αSYN transgenic mice and analysed possible neuroprotective effects and the capacity of modulating neuroinflammation.

## Methods

### Animals

In the present study homozygous (PLP)-α-SYN mice [24] at the age of 18 months were used. The animal study was designed compliant with the Austrian guidelines for the care and use of laboratory animals and all experiments were approved by the Federal Ministry for Education, Science and Research of Austria with the reference number do. ZI. 5004. Animals were housed at the Animal Facility of the Innsbruck Medical University under a 12-hour light/dark cycle with food and water available *ad libitum*.

### Isolation of GFP MSCs

MSCs were obtained from C57BL/6-Tg(UBC-GFP)30Scha/J mice (Charles River, Germany), 6–8 weeks old, expressing the enhanced green fluorescent protein (GFP) gene under the human ubiquitin C promoter [25]. Primary GFP mMSC cultures were isolated according to established protocols [26].

Briefly, tibia and femur were treated with collagenase (Sigma, St. Louis, MO, USA) for 2 h, 37°C, 20% O_2_, 5% CO_2_. Thereafter, fragmented bones were centrifuged and cell fractions were loaded on a Ficoll-Paque Plus gradient (Amersham Biosciences, Piscataway, NJ, USA) to harvest cells from the interphase, followed by a washing step. The isolated bone marrow cells were seeded for expansion in complete isolation medium constituted of RPMI-1640 (Gibco, Invitrogen, Carlsbad, Ca, USA) supplemented with 20% fetal bovine serum (Invitrogen), 100 units/ml penicillin and 100 µg/ml streptomycin (Invitrogen). After 24 hours non-adhering cells were removed by extensive washing with Dulbecco's phosphate buffered saline (DPBS Invitrogen). The attached cells were cultured until confluent and subsequently subcultured at low density (50 cells/cm^2^) with complete expansion medium consistent of Iscove's Modified Dulbecco's Medium (IMDM, Invitrogen) supplemented with 20% fetal bovine serum (Invitrogen), 100 units/ml penicillin and 100 µg/ml streptomycin (Invitrogen). Medium exchange was performed twice weekly.

### Characterization of GFP MSCs by flow cytometry

GFP MSCs were washed with DPBS, harvested with 0.25% trypsin and 1 mM EDTA (Invitrogen) for five minutes at 37°C, divided into round-bottom polystyrene tubes and incubated with pooled mouse IgG (Sigma Aldrich, St. Louis, MO, USA) for 15 minutes at room temperature (RT). Subsequently, cells were labelled with phycoerythrin (PE)- or peridinin-chlorophyll protein complex (PerCP)-conjugated monoclonal antibodies (mABs) specific for CD29, CD11b, CD105, CD34, CD117 (c-kit), CD44, Ly6A/E (Sca-1) (all Biolegend, San Diego, CA, USA) as wells as SSEA-4 (R&D Systems, Mineapolis, MN, USA), MHC Class II (I-A/I-E), MHC Class I (H2D) and CD45 (all Becton Dickinson Biosciences, San Jose, CA, USA) for 30 minutes at 4°C in the dark. PE- as well as PerCP-conjugated isotype-matched mABs were used as negative controls. To asses cell viability Via**-**Probe™ Cell Viability solution (Becton Dickinson Biosciences, San Jose, CA, USA) was added shortly before flow cytometric analysis. Two additional washes were performed and cell surface antigen expression was analyzed on a FACScan using CellQuest^TM^ software (both BD Biosciences, San Jose, CA, USA) with 10,000 events recorded for each sample.

### In vitro differentiation of GFP MSCs

Assessing the potential of isolated cells to differentiate into osteogenic and adipogenic lineages was performed as previously described [27]. Briefly osteogenic differentiation was induced culturing MSCs in 6-well culture plates (TPP, Trasadingen, Switzerland) in IMDM medium containing 10% FBS, 100 units/ml penicillin and 100 µg/ml streptomycin and supplemented with 50 µM ascorbate 2-phosphate, 10 mM β-glycerol phosphate and 100 nM dexamethasone (all from Sigma Aldrich, St. Louis, MO, USA). Medium was changed twice a week for a period of 2–3 weeks. To observe calcium deposition, cultures were washed with PBS, fixed with 4% paraformaldehyde (PFA, Sigma Aldrich, St. Louis, MO, USA) for ten minutes and stained with Alizarin Red, pH 4.1, for ten minutes on a rotating platform. Cultures were rinsed two or three times with PBS to reduce non-specific staining.

Adipogenic differentiation was induced after growing MSCs as a monolayer and allowing them to become confluent. Complete medium was exchanged to adipogenic induction medium consisting of IMDM medium containing 10% FBS, 100 units/ml penicillin and 100 µg/ml streptomycin, 1 µM dexamethasone and 0.5 mM methyl-isobutylxanthine, 10 µg/ml insulin and 100 µM indomethacin (all from Sigma Aldrich, St. Louis, MO, USA). Cells were incubated in this medium 48–72 hours and then adipogenic maintenance medium containing 10 µg/ml insulin and 10% FBS in IMDM was applied for 24 hours. Cells were then again treated for 48–72 hours with adipogenic induction medium followed by a period of 24 hours in maintenance medium and a third treatment with induction medium. Finally, cultures were kept for one week in adipogenic maintenance medium. Cells were fixed in 4% PFA and lipid droplet staining was performed using Oil Red O (Sigma Aldrich, St. Louis, MO, USA).

### Cell transplantation

For MSC transplantation, two groups of (PLP)-α-SYN mice were included in the study, one group termed (PLP)-α-SYN+MSC (n = 12) receiving 500,000 cells in 150 µl of saline through the tail vein, and the control group termed (PLP)-α-SYN (n = 6) sham injected with an equal amount of saline only. Survival in the (PLP)-α-SYN+MSC group was around 60%.

### Behaviour

To determine the efficacy of intravenously transplanted MSCs with respect to a potential amelioration of motor dysfunction in the (PLP)-α-SYN mouse modelling MSA [28], the following motor function assessment was carried out: beam walking test and stride length analysis with DigiGait.

#### Beam walking test

Fine motor coordination and balance capabilities of mice were assessed by the beam walking test [29]. The beams consisted of long stripes of wood (each measuring 70 cm) with square cross sections of 0.9 cm and 1.6 cm, horizontally placed 50 cm above the bench surface. The mice were encouraged to walk a distance of 50 cm. For training, three daily sessions of three trials (9 crossings) were performed using the 1.6 cm square large beam. Mice were then tested 1 week, 2 weeks and 4 weeks post transplantation (p.t.) using the 0.9 cm square beam. Mice were allowed up to 60 seconds to traverse the beam. The latency to traverse the beam and the number of times the hind feet slipped off, over the given distance of 50 cm, were recorded for three consecutive runs. Analysis of each session was based on the mean score of the three trials.

#### Stride length analysis with DigiGait

The DigiGait System (DigiGait Imaging System, Mouse Specifics, Boston, MA, USA) is a non-invasive method for quantitatively compare gait dynamics [30]. Each mouse was placed on a transparent belt of a treadmill enclosed by a plastic scaffold. The speed of the treadmill was set to 20 cm/s for all experimental groups. The ventral side of the mice as they walk was imaged by a high-speed camera, which captured the dynamics of the paws and corresponding limbs as they approach and move away from the belt. A special software, DigiGait Imaging System, Mouse Specifics, Boston, MA, USA) automatically calculated stride length and other spatial and temporal gait indices for each limb. DigiGait analysis was performed at 1 week, 2 and 4 weeks p.t.

### Tissue processing

Four weeks after MSC treatment, animals were transcardially perfused with PBS under deep thiopental anaesthesia. Brains were removed and cut to separate the hemispheres. One hemisphere was put into 4% PFA overnight and cryoprotected with 20% sucrose. Brains were slowly frozen and kept at −80°C for further processing.

The other hemisphere was cut to obtain midbrain-brainstem tissue, put into a cryovial (Nunc, Rochester, New York, USA) and frozen in liquid nitrogen and stored at −80°C until use. A modified RIPA buffer [31] was used to homogenize brain tissue of each preparation. Brain homogenates were centrifuged at 16,000 g for ten minutes at 4°C and supernatants were stored at −80°C until further processing.

### Immunohistochemistry

Six series of 40 µm sections throughout the whole hemisphere were cut on a cryostat (Leica, Nussloch, Germany). One series was directly mounted on gelatine-coated slides and used for cresyl violet (Nissl) staining. Immunolabelling was performed on free floating sections using the following antibodies: rat anti-mouse CD11b (1∶150; AbD Serotec, Oxford, UK), polyclonal rabbit anti-green fluorescence protein (1∶1000; GFP, Abcam, Cambridge, UK), polyclonal rabbit CD3 (1∶7500, Abcam, Cambrige, UK) and monoclonal mouse anti-tyrosine hydroxilase (TH, 1∶1000, Sigma, St. Louis, MO, USA). Secondary antibodies were biotinylated anti-rat IgG, biotinylated anti-rabbit IgG, anti-mouse IgG (1∶200, all Vector Laboratories, Burlingame, CA), Alexa-fluor 488-conjugated goat anti-rabbit or Alexa-fluor 594-conjugated goat anti-rat (both 1∶500, Molecular Probes, Leiden), respectively.

Endogenous peroxidase activity was quenched in H_2_O_2._ After normal serum blocking, sections were incubated with the primary antibody overnight at 4°C, followed by incubation in biotinylated secondary antibody. After incubation in Vectastain ABC reagent (Vectastain ABC kit, Vector Laboratories, Burlingame, CA), the immunohistochemical reaction was developed with 3,3′-diaminobenzidine (DAB) and sections were mounted onto gelatine-coated slides, counterstained with cresyl violet or Mayer's haematoxylin solution, dehydrated and coverslipped with Entellan. Immunofluorescence staining performed for tracing GFP positive MSCs was carried out by normal serum blocking and overnight incubation with the anti-GFP primary antibody followed by incubation with the respective secondary antibody, and counterstaining of the nucleus by 4′, 6-Diamidin-2′-phenylindoldihydrochlorid (DAPI, Sigma Aldrich, St. Louis, MO, USA). For GFP staining, brain sections from C57BL/6-Tg(UBC-GFP)30Scha/J mice were used as positive controls and tissue sections from (PLP)-αSYN transgenic animals as negative controls. Double-immunofluorescence staining for GFP and CD11b was performed as described above; dilution of the commercial rat anti-mouse CD11b antibody was 1∶50.

### Quantification of α-synuclein concentration in brain lysates

Brain lysates were analysed for αSYN concentration using an α-synuclein immunoassay kit (Invitrogen, Carlsbad, CA, USA), following manufacturer's instructions. ELISA plates were analysed with a multi-well plate reader at 450 nm (Beckman Coulter, Brea, CA, USA).

### Quantification of cytokine concentrations in brain lysates

Brain lysates were analysed using the mouse Th1/Th2 10-plex kit, MCP-1 and TGF-β1 (Flow Cytomix, Bender MedSystems, Vienna, Austria) according to the manufacturer's instructions. Data were acquired using a FACScan (BD Biosciences, San Jose, CA, USA) with 1500 events recorded for each sample and further analysed by the Flow Cytomix Software version 2.3 (Bender MedSystems, Vienna, Austria).

### Microscopy and image analysis technique

Cell culture microscopy was performed using a Leica DMI 4000B microscope and Application Suite V3.1 (Leica, Wetzlar, Germany). Fluorescent histological sections were analysed with the aid of an ApoTome® microscope and AxioVision Software (both Carl Zeiss Microimaging GmbH, Jena, Germany).

All morphometric analysis was done in a blinded way applying a computer-assisted image analysis system (Nikon E-800 microscope, CCD video camera, Optronics MicroFire, Goleta, USA; Stereo Investigator Software, MicroBrightField Europe e.K., Magdeburg, Germany). The optical fractionator method [32], [33] was used to estimate the total number of neurons and microglia in the substantia nigra pars compacta (SNc).

### Statistics

All data are given as means ± standard error of the mean (SEM). Behavioural data were compared by two-way analysis of variance (ANOVA) for time and treatment effects followed by a *post hoc* Bonferroni test (corrected for multiple comparisons). Data from cytokine measurements were subjected to two-tailed unpaired Student's *t-test* with regard to treatment. Data obtained from image analysis technique were analysed by unpaired Student's *t-test*. Correlations between cytokines and the number of TH^+^ neurons were performed with the Pearson correlation analysis. All statistical analyses were performed with GraphPad Prism 5 Software (GraphPad Software Inc., San Diego, CA, USA). A p-value of p<0.05 was considered significant.

## Results

### Isolation and characterization of murine GFP MSCs

MSCs of GFP transgenic mice were isolated from tibia and femur and kept in culture for several passages. For characterization, differentiation and transplantation, cells at passage 8 were used (**Figure S1, A**). Before intravenous application the cells were characterised, flow cytometry analysis confirmed that the cells at the stage of transplantation were positive for GFP (96.65%), CD29 (98.72%), CD44 (99.51%), CD105 (99.43%), MHC Class I (H2D, 48.23%), Sca-1 (99.48%), SSEA-4 (95.91%) and had a low number of CD11b (13.28%), CD34 (13.10%), CD45 (1.72%), CD117 (c-kit, 15.76%) and MHC Class II (I-A/I-E, 11.84%). At the stage of transplantation 99.92% of all cells were viable, as revealed by Via Probe staining.

Multilineage potential was demonstrated by differentiation into adipocytes, indicated by Oil-Red O staining (**Figure S1, B**) as well as calcium deposits indicating osteogenic lineage differentiation when stained with Alizarin Red (**Figure S1, C**).

### Cell transplantation and tracing of GFP MSCs

**Figure 1:**
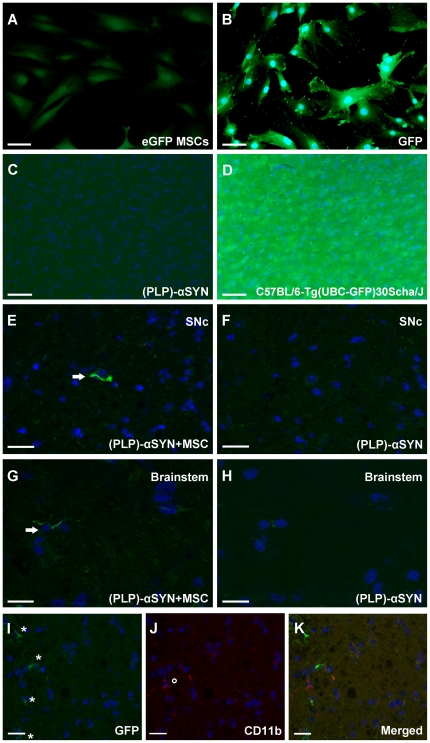
Tracing of intravenously infused GFP^+^ MSCs in aged (PLP)-αSYN transgenic mice. (**A**) GFP^+^ MSCs in culture showed diffuse green fluorescence which could be further enhanced by immunocytochemistry using GFP antibody (**B**). To control the same GFP antibody in brain sections, negative- ((PLP)-αSYN mice, **C**) and positive (C57BL/6-Tg(UBC-GFP)30Scha/J transgenic mice, **D**) tissue sources were applied. Scale bars (**A**, **B**, **C**, **D**) represent 50 µm. Single GFP^+^ MSCs could be observed, throughout the brain in transplanted animals, by ApoTome confocal-like images of SN (**E**) and brainstem (**G**). Negative control sections of GFP staining in (PLP)-αSYN mice of SN (**F**) and brainstem (**H**). Scale bars (**E**, **F, G, H**) represent 20 µm. Arrows indicate GFP^+^ MSCs. ApoTome confocal-like images of GFP (**I**) and CD11b positive cells (**J**) in PLP-αSYN+MSC mice. The merged image (**K**) indicates that GFP^+^ cells do not colocalize with CD11b^+^ cells. Asterisk indicate GFP^+^ cells, circle indicates CD11b^+^ cell. Scale bars (**I**, **J**, **K**) represent 20 µm. GFP, green fluorescent protein; SN, substantia nigra.

Two groups of aged (PLP)-α-SYN mice were included in the study, one group receiving 500,000 cells/150 µl of saline through the tail vein designated (PLP)-αSYN+MSC, and one group serving as controls with an equal amount of saline only into the tail vein termed (PLP)-αSYN. Immunofluorescence with a polyclonal rabbit anti-GFP antibody was performed on PFA fixed MSCs in culture (**Figure 1A**) to ensure tracing of GFP MSCs (**Figure 1B**). Single engrafted donor cells were detected four weeks post MSC injection in the (PLP)-αSYN+MSC group (**Figure 1E and 1G**) whereas sections from PLP-αSYN mice served as negative controls (Figure **1F and 1H**). To control the efficiency of the antibody in fixed tissue, brain sections from (PLP)-αSYN control group served as negative control (**Figure 1C**) while brain sections from C57BL/6-Tg(UBC-GFP)30Scha/J served as positive (**Figure 1D**) control. In face of potential contaminations of myeloid cells (CD11b) in the MSC culture, double staining with GFP and CD11b **(** **Figure 1I, J and K**) was performed in sections of the (PLP)-αSYN+MSC group to prove the phenotype of GFP positive cells being MSCs and not myeloid cells.

### Behaviour

**Figure 2:**
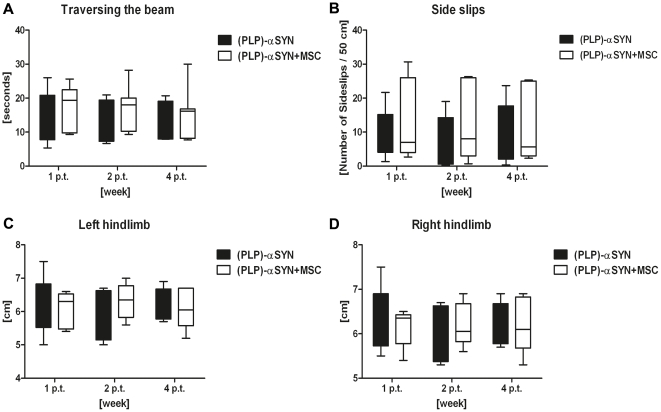
Assessing motor coordination with the beam walking test and DigiGait analysis. Duration to traverse a narrow beam analysed 1 week, 2 weeks and 4 weeks post transplantation (p.t.) (**A**). No difference between (PLP)-αSYN (n = 6) and (PLP)-αSYN+MSC (n = 7) treated animals was found (p>0.05). Number of side slips traversing a 50 cm narrow beam was analysed at 1 week, 2 weeks and 4 weeks p.t. (**B**). Statistical analysis did not reveal any significant difference between both groups. Stride length analysis of left (**C)** and right (**D**) hindlimbs of (PLP)-αSYN+MSC (n = 7) versus (PLP)-αSYN controls (n = 6), 1 week, 2 weeks and 4 weeks p.t. Stride length did not significantly alter in MSC treated animals compared to saline treated controls (p>0.05). Data were analysed by two-way ANOVA, following Bonferroni *post-hoc* test (for multiple corrections) and are presented as means ± SEM. Statistical significance was set at p<0.05.

The efficacy of intravenously transplanted MSCs to restore motor function in aged (PLP)-αSYN mice versus controls, was measured with the beam walking test, and stride length was analysed with the DigiGait system. The beam walking test determines fine motor coordination and balance capabilities. Traversing the beam was performed 1 week, 2 and 4 weeks p.t. Over the period of 4 weeks, no significant improvement in the time traversing the beam (**Figure 2A**) (p>0.05) or in the number of sideslips (**Figure 2B**) (p>0.05) was detected.

We analysed the stride length on both hindlimbs (left, right) with the DigiGait System in the transplant and control group. Previous results from our research group have demonstrated that (PLP)-αSYN transgenic mice show shortening of hindlimb stride length associated with TH^+^ cell loss in the SNc [28]. In the current study we analysed the stride length of the left (**Figure 2C**) and right (**Figure 2D**) hindlimbs in the transplant and control group at 1 week, 2 week and 4 weeks p.t. Stride length in animals treated with i.v. MSCs was not significantly altered from stride length in the control group (for both hindlimbs p>0.05).

Both tests demonstrate that i.v. MSC treatment in aged (PLP)-αSYN mice has not induced changes in motor behaviour.

### Neuroprotective effect on TH^+^ neurons in the SNc after MSC treatment

**Figure 3:**
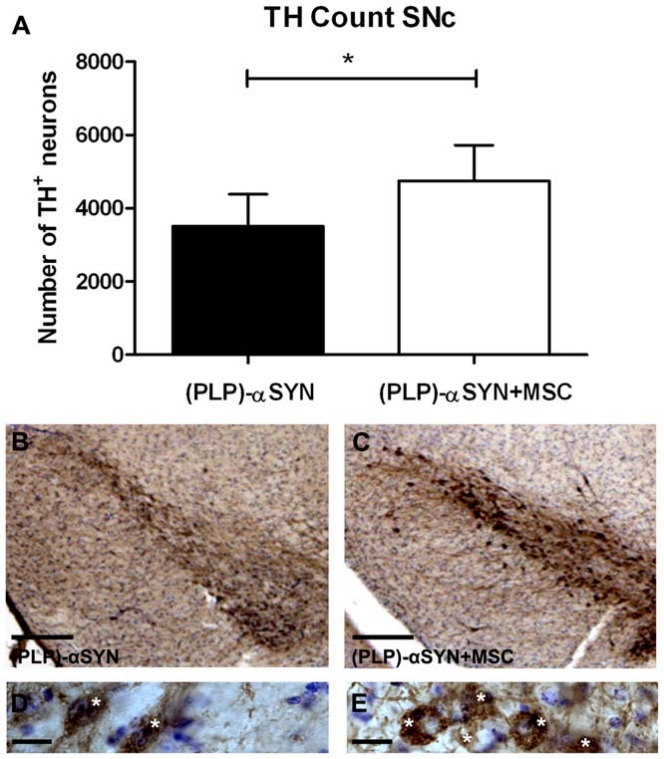
Neuroprotection of dopaminergic neurons in SNc after MSC treatment of (PLP)-αSYN mice. Quantification of TH-immunoreactive neurons in the SNc demonstrating a significant survival of TH^+^ neurons (p = 0.036) in animals with MSC treatment compared to controls, four weeks after i.v. MSC transplantation (**A**). Low magnification of TH immunohistochemistry, counterstained with Nissl in (PLP)-αYN (**B**) and (PLP)-αSYN+MSC treated animals (**C**). Scale bar (**A**,**B**) represent 300 µm. High magnification of TH neurons in SNc of Tg (**D**) and Tg+MSC treated animals (**E**). Scale bar (**D**,**E**) represent 20 µm. Cell counts were analysed by unpaired Student's *t-test* and the level of significance was set at p<0.05 (* p<0.05). Data are presented as means ± SEM. (PLP)-αSYN (n = 6); (PLP)-αSYN+MSC (n = 7); Asterisk in **D** and **E** indicate TH^+^ neurons.

Reduction of TH-immunoreactive neurons was previously reported in the SNc of (PLP)-αSYN transgenic mice [28] suggesting that the presence of αSYN in oligodendrocytes induces dopaminergic neuron loss. We performed TH staining and stereological counting in the SNc in the MSC treatment and control group **(** **Figure 3A–3E** **)** demonstrating a significant recovery of the total number of TH^+^ neurons in the MSC treated group ((PLP)-αSYN+MSC 4747±356.8 vs. (PLP)-αSYN 3510±368.8; p = 0.036) 4 weeks after transplantation.

### αSYN concentration in midbrain-brainstem lysates

**Figure 4:**
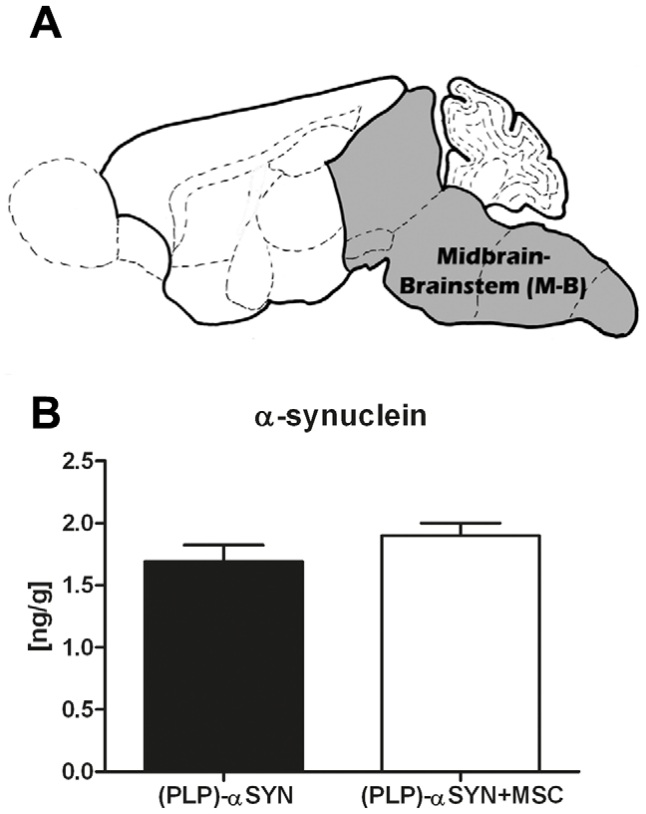
αSYN concentration is not altered in midbrain-brainstem lysates of MSC treated mice. αSYN concentrations in midbrain-brainstem lysates (**A**) were determined for the (PLP)-αSYN (n = 6) and (PLP)-αSYN+MSC (n = 7) group by ELISA. Student's unpaired *t-test* revealed no significant difference between both groups (p = 0.237) (**B**). Data are presented as means ± SEM with statistical significance p>0.05.

In previous studies, research on the (PLP)-α-SYN mouse model has demonstrated that pathological αSYN accumulation promotes degeneration of neurons in the SNc, locus coeruleus, nucleus ambiguous, laterodorsal tegmental nucleus, pedunculopontine nucleus and Onuf's nucleus [34], [35] similar to findings in MSA patients (reviewed in [2]). With a αSYN immunoassay we investigate whether MSC treatment had an effect on αSYN concentration in midbrain-brainstem lysates. We chose midbrain-brainstem samples (**Figure 4A**) since the dissected area includes the affected nuclei. Statistical analysis by unpaired Student's *t-test* did not show differences on αSYN concentration in MSC treated (n = 7) versus control animals (n = 6) ((PLP)-α-SYN+MSC 1.899±0.1032 vs. (PLP)-α-SYN 1.692±0.1322, p = 0.237) (**Figure 4B**).

### MSCs influence cytokine levels in midbrain-brainstem lysates

**Figure 5:**
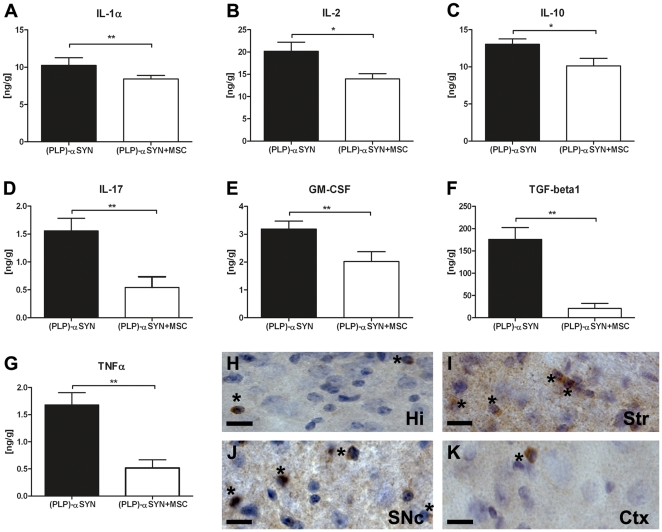
Influence of MSC treatment on cytokine concentrations in midbrain-brainstem lysates. Cytokine levels in midbrain-brainstem lysates were quantified by flow cytometry. Significant downregulation of IL-1α (p = 0.0014) (**A**), IL-2 (p = 0.019) (**B**), IL-10 (p = 0.046) (**C**), IL-17 (p = 0.056) (**D**), GM-CSF (p = 0.029) (**E**), TGF-β1 (p = 0.001) (**F**) and TNFα (p = 0.001) (**G**) in MSC treated animals (n = 7) versus controls (n = 6) was observed. All data are presented as means ± SEM and were analysed by the Student's unpaired *t-test*. (* p<0.05; ** p<0.01; *** p<0.001). Representative CD3^+^ stainings counterstained with Mayer's haematoxylin solution demonstrated invading T-cells throughout the brain, as presented here in hippocampus (**H**), striatum (**I**), substantia nigra pars compacta (**J)** and cortex (**K**) in (PLP)-αSYN transgenic animals. A similar staining pattern was observed in (PLP)-αSYN+MSC treated animals. Scale bar (**H**, **I**, **J**, **K**) represents 10 µm. Asterisk indicate CD3^+^ T-cells. Hi, hippocampus; Str, striatum, SNc, substantia nigra pars compacta; Ctx, cortex.

IL-1α, IL-2, IL-4, IL-5, IL-6, IL-10, IL-17, GM-CSF, INFγ, MCP-1, TGF-β1 and TNFα were assessed in midbrain-brainstem lysates of (PLP)-αSYN+MSC versus (PLP)-αSYN animals. Four weeks after intravenous MSC application, we observed a significant downregulation of IL-1α (p = 0.0014), IL-2 (p = 0.019), IL-10 (p = 0.046), IL-17 (p = 0.056), GM-CSF (p = 0.029), TGF-β1 (p = 0.001) and TNFα (p = 0.001) (**Figure 5A - 5G**) in midbrain-brainstem lysates of MSC treated animals versus control group, whereas no significant difference was found for IL-4 (p = 0.16), MCP-1 (p = 0.18), IL-5 (0.072), IL-6 (p = 0.18) and INFγ (p = 0.31).

Due to a significant reduction of the lymphocytic signalling molecules IL-2 and IL-17, we performed a staining for the T-cell marker CD3 in (PLP)-αSYN+MSC and (PLP)-αSYN animals. We could detect single CD3^+^ cells throughout different brain regions (**Figure 5H–5K**).

Moreover, we analysed microglia as an additional source of cytokines. Previous work of our group has shown that microglial activation in the (PLP)-αSYN mouse is present in the SNc and mediates neurodegeneration [34]. Hence counting CD11b-immunoreactive cells in the SNc as a marker for microglia was performed (**Figure S2, B–C**). Statistical analysis with two-tailed unpaired Student's *t-test*, revealed no significant difference in the number of CD11b^+^ cells in transplanted animals (n = 7) versus the control group (n = 6) ((PLP)-αSYN+MSC 6588±242 vs. (PLP)-αSYN 6880±529.3; p = 0.6075) (**Figure S2, A**).

Finally, in order to evaluate whether the cytokines in midbrain-brainstem lysates (IL-1α, IL-2, IL-4, IL-5, IL-6, IL-10, IL-17, GM-CSF, INFγ, MCP-1, TGF-β1 and TNFα) contribute to TH^+^ neuronal rescue we established a Pearson correlation between the cytokines and TH^+^ neurons (**Table 1**). We could demonstrate significant inverse correlations for IL-1α (Pearson r = −0.697, p = 0.0081), IL-2 (Pearson r = −0.823, p = 0.0005), TGF-β1 (Pearson r = −0.647, p = 0.0169) and TNFα (Pearson r = −0.0575, p = 0.0397).

**Table 1:** Correlation between cytokines and TH neurons.

Cytokine	Pearson r	p-value	Significance
**IL-1α**	−0.6970	0.0081	**
**IL-2**	−0.8234	0.0005	***
**IL-4**	−0.1205	0.6950	n.s.
**IL-5**	−0.4112	0.1628	n.s.
**IL-6**	−0.5122	0.0735	n.s.
**IL-10**	−0.5149	0.0718	n.s.
**IL-17**	−0.3720	0.2106	n.s.
**GM-CSF**	−0.3958	0.1806	n.s.
**INFγ**	−0.01089	0.9718	n.s.
**MCP-1**	−0.4772	0.0992	n.s.
**TGF-β1**	−0.6465	0.0169	*
**TNFα**	−0.5752	0.0397	*

**Abbreviations:** interleukin-1alpha (**IL-1α**), interleukin-2 (**IL-2**), interleukin-4 (**IL-4**), interleukin-5 (**IL-5**), interleukin-6 (**IL-6**), interleukin-10 (**IL-10**), interleukin-17 (**IL-17**), granulocyte macrophage colony-stimulating factor (**GM-CSF**), interferon gamma **(INFγ**), monocyte chemotactic protein-1 (**MCP-1**), transforming growth factor-beta1 (**TGF-β1**), tumor necrosis factor alpha (**TNFα**);

*p<0.05,

**p<0.01,

***p<0.001;

not significant (n.s.).

## Discussion

In the present study we aimed to determine whether intravenous application of murine MSCs in an aged (PLP)-αSYN MSA mouse model ameliorates behavioural deficits and exerts neuroprotective and immunomodulatory properties. To date, to our knowledge there is no experimental evidence of a MSC therapeutic approach in transgenic MSA models. Recently, human MSCs have been demonstrated to protect against loss of neurons in SN and striatum in an animal model of double toxin-induced MSA-P [23]. A few years ago, in an open-label study design, disease modifying effects of intra-arterial and intravenous injected MSCs in eleven patients with MSA-C, have been reported [22]. Together with *in vitro* and *in vivo* findings in PD and other neurodegenerative disorders, MSCs seem to be an attractive and feasible therapeutic intervention [20], [23], [36]–[39].

As a matter of fact, experimental results are often difficult to compare, in lieu of standards for MSC isolation, cultivation and in vivo application. Furthermore there is no unique marker to identify MSCs. In general, MSCs are characterised upon expression of a group of surface receptors and upon their multilineage potential [40]. Prior to transplantation we have characterized GFP MSCs, based on a set of criteria proposed by the International Society for Cellular Thearpy [41], which includes expression and lack of surface markers and differentiation potential into adipocytes and osteocytes.

In the (PLP)-αSYN+MSC transplant group the survival rate after intravenous MSC infusion was low, probably due to the high age of the animals. Further we presume that due to their size MSCs got trapped within the pulmonary capillaries, causing pulmonary and hemodynamic alterations [42]. On the other hand numerous animal studies and clinical trials have reported favourable outcomes following systemic infusion of MSCs [36], [43].

Since we have only encountered a few single donor engrafted GFP^+^ MSCs four weeks after transplantation, the use of GFP as experimental tool to examine the survival and fate in future studies is in question, since there are a lot of inconsistent results in the literature, on tracking and determining cell fate of MSCs using GFP as a reporter in transplantation studies [44], [45]. In addition, we cannot exclude a detrimental effect of the GFP protein on MSC survival and therefore preventing proper integration into sites of neurodegeneration. On the other hand, a number of reports state that MSCs exert effects on tissue repair despite exhibiting low and/or transient levels of engraftment [20], [40]. This foreshadows a novel concept of tissue repair relying on secretion of trophic factors and/or crosstalk with the microenvironment rather than MSC transdifferentation.

The (PLP)-αSYN transgenic mouse model has been widely characterized in terms of effects of αSYN overexpression on neurodegeneration and motor activity [28], [35]. Furthermore, this animal model has been successfully applied in neuroprotective studies as a preclinical rationale for phase II clinical trials [46], [47]. We performed the beamwalking test to define fine motor coordination and balance capabilities as well as stride length with a digital system, since shortening of the stride length has been reported in the (PLP)-αSYN transgenic mouse [28]. In a recent study, intravenous human MSCs have ameliorated behavioural deficits in a double-toxin induced mouse model of MSA-P [23]. Our experimental data show no significant effects of intravenously applied MSCs to alleviate behavioural failure. On the other hand, the study with double-toxin induced striatonigral degeneration has been performed in mice lacking αSYN expression in oligodendroglia and thus replicating solely striatonigral-like pathology without reproducing other cardinal features of MSA. It remains elusive whether oligodendroglial αSYN exerts a deleterious effect on transplanted MSCs, however in a recent study addressing the fate of embryonic striatal grafts in presence of oligodendroglial αSYN inclusions, disturbed dopaminergic re-innervation and reduced p-zone volume of the grafts in the MSA mouse model has been attributed to effects of host αSYN pathology [48].

For our pilot study, we have chosen transgenic animals at the age of 18 months, overexpressing αSYN under control of the PLP promoter, since GCIs are the hallmark of MSA and MSA is a late onset disease [49], [50]. Previous studies have clearly demonstrated that in this MSA animal model αSYN overexpression leads to neurodegeneration [28], [35] resembling human neuropathology. At the age of 18 months, neurodegeneration due to αSYN overexpression is at a much more progressed stage, impairing motor activity drastically as shown in MSA mouse models overexpressing αSYN under control of oligodendroglial promoters [51], [52]. Currently we are examining this issue more closely in the (PLP)-αSYN mouse. Nevertheless, the absent behavioural improvement after MSC treatment, leads to the conclusion, that MSC treatment at later disease stages does not induce the desired effect of ameliorating behavioural deficits.

Since one pathological feature in MSA patients covers selective neuronal loss in the SNc, and the (PLP)-αSYN mouse model replicates this feature, we evaluated whether MSC treatment had an effect on number of TH^+^ neurons. There was a subtle but significant recovery of numbers of dopaminergic neurons in the MSC transplant group compared to transgenic controls.

We further investigated putative factors that may contribute to this “rescue” of dopaminergic neurons after MSC treatment. We analysed whether TH recovery is caused by the decrease of αSYN concentration in midbrain-brainstem lysates. αSYN is known to be a key factor involved in oligodendroglial and neuronal loss in MSA patients and in the (PLP)-αSYN transgenic animal model [3], [28], [34], [35]. Recently genetic variants in the αSYN gene *SNCA* have been associated with an increased risk in developing MSA [53], [54]. However, αSYN concentration in the midbrain-brainstem region was not significantly altered between (PLP)-αSYN+MSC treatment compared to (PLP)-αSYN control group. This finding highlights the concept of tissue repair of MSCs by releasing anti-inflammatory and trophic molecules.

Neuroinflammation has been widely regarded as a possible key player in progressing disease pathogenesis in various neurodegenerative diseases. In PD patients as well as PD animal models, neuroinflammation in terms of microglial activation has been observed [9]. Lately emerging evidence for the presence of T-lymphocytes in the midbrain of PD patients suggests that a potential role of infiltrated peripheral cells is related to PD pathogenesis [9], [55]. In a recent study infiltration of T-cells into the brain actively participated in dopaminergic neuron degeneration in the SNc [56]. Additionally, overexpression of human αSYN in mouse SN neurons, induced by an adeno-associated viral vector, has led to activation of microglia, production of inflammatory cytokines and stimulated the adaptive immune response [57]. In our experiment we analysed twelve cytokines, IL-1α, IL-2, IL-4, IL-5, IL-6, IL-10, IL-17, GM-CSF, INFγ, MCP-1, TGF-β1 and TNFα in midbrain-brainstem lysates and found significant downregulation of IL-1α, IL-2, IL-10, IL-17, GM-CSF, TGF-β1 and TNFα four weeks after intravenous MSC application in the treatment group. Additionally we encountered CD3^+^ T-cells throughout the brain in (PLP)-αSYN and (PLP)-αSYN+MSC treated animals. After MSC treatment, however, the T-cell specific cytokines IL-2 and IL-17 were significantly downregulated. Furthermore, TH neuronal “rescue” was inversely correlated with IL-2, indicating that MSC treatment influenced pathogenic T-cell response in (PLP)-αSYN mice. Similar effects have been widely investigated for multiple sclerosis and experimental autoimmune encephalitis and support the role of MSC treatment by modulation of T-cell response [58].

Finally we analyzed the influence of MSC treatment on microglial activation, since microglia have been reported to parallel the neuronal multisystem degeneration in MSA [8] and mediates dopaminergic neuronal loss related to oligodendroglial α-synucleinopathy in the (PLP)-αSYN mouse [34]. We quantified the number CD11b^+^ microglial cells in the SNc, yet could not demonstrate a significant difference in microglial cell number after MSC treatment. However, this finding cannot exclude a modulatory effect on microglial activation status. Furthermore, the major proinflammatory cytokine TNFα and IL-1α, also released by activated microglia and astroglia was significantly decreased in brain lysates of the MSC treatment group and inversely correlate with the number of TH neurons indicating that suppression of microglial activation and astrogliosis may contribute to dopaminergic neuronal survival. Similar results, demonstrating decreased activation of astrocytes and microglia by human MSCs in a mouse model of MSA-P have been recently reported [23] and are in good concordance with our findings that MSCs exert modulatory effects on neuroinflammation and promote survival of dopaminergic neurons in the SNc.

In summary, our study describes the first experimental attempt using MSCs as a therapeutic intervention in an aged transgenic mouse model of MSA featuring oligodendroglial α-synucleinopathy. We have demonstrated that intravenous application of MSCs leads to a rescue of dopaminergic neurons in the SNc. Furthermore we could demonstrate a profound immunomodulatory effect after MSC treatment, resulting in downregulation of various proinflammatory cytokines, which are linked to microglial activation, astrogliosis and mediation of adaptive immunity. However, MSC treatment did not alter behavioural deficits in aged transgenic MSA mice.

Our data have potential implications for MSCs as a future stem cell source in MSA therapies. Nevertheless, further experimental studies on the efficacy of MSCs as disease modifying candidates in MSA as well as different routes of application have to be performed. Prior to embarking on further human trials, preclinical studies are necessary since they will reveal if all that glitters experimentally is truly clinical gold.

## Supporting Information

Figure S1 **Differentiation of MSCs into adipocytes and osteocytes.** After applying lineage specific induction media, MSCs (**A**) differentiated into adipocytes, demonstrated by presence of Oil-Red O lipid droplet staining (**B**). Differentiation into the osteogenic lineage was demonstrated by Alizarin Red staining, identifying calcification of cells (**C**). Scale bar (**A**) represents 20 µm, scale bars (**B**,**C**) represent 50 µm.
(TIF)

Figure S2 **MSC treatment had no effect on the number of CD11b^+^ microglial cells.** Quantification of CD11b^+^ microglial cells by stereological counting demonstrated that four weeks after MSC treatment the number of CD11b^+^ cells was not significantly altered in the treatment group compared to controls (p = 0.6075) (**A**). All data are presented as means ± SEM and were analysed by unpaired Student's *t-test*. Immunohistochemical staining of CD11b^+^ microglial cells in the SNc of a (PLP)-αSYN (n = 6) (**B**) and (PLP)-αSYN+MSC (n = 7) mouse (**C**). Scale bars (**B**,**C**) represent 20 µm. Asterisk indicate CD11b^+^ cells.
(TIF)
