# Supplementary figures and images for: Mesenchymal Stem Cells in a Transgenic Mouse Model of Multiple System Atrophy: Immunomodulation and Neuroprotection
Source: PLoS One. 2011 May 18;6(5):e19808. doi: 10.1371/journal.pone.0019808 (PMC3097217; doi:10.1371/journal.pone.0019808)

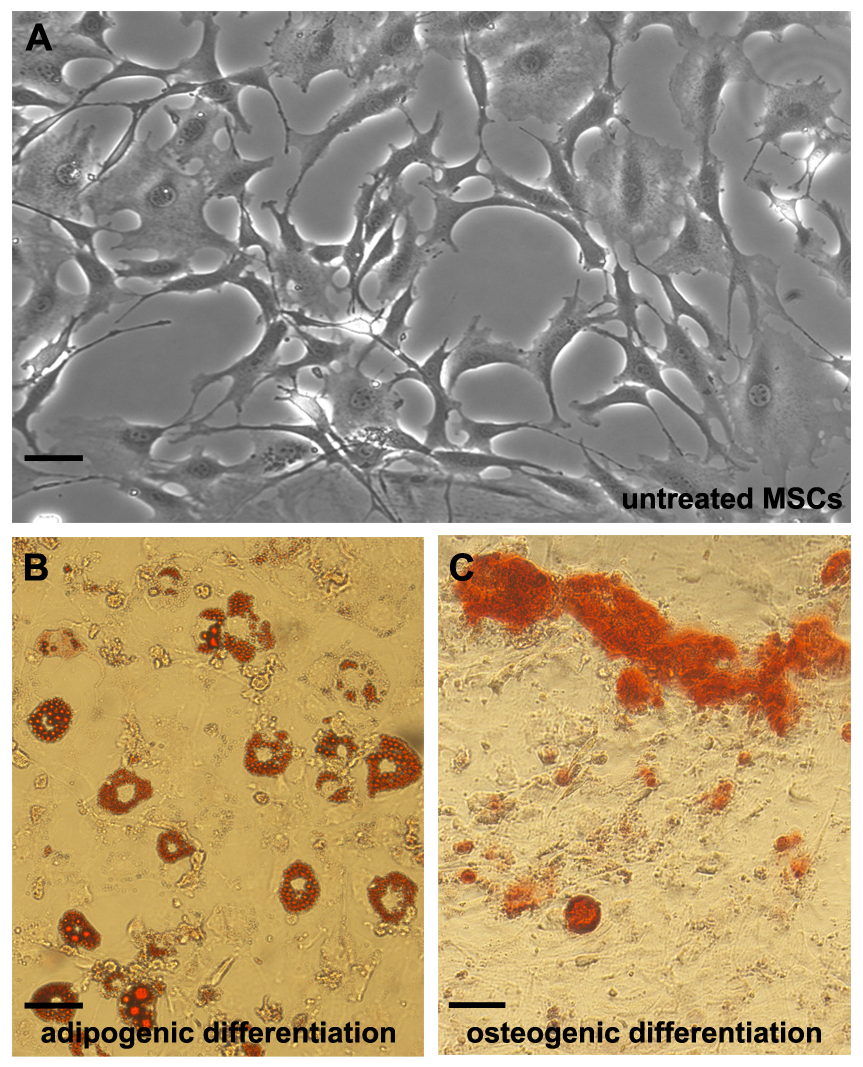

Supplement: Figure S1 — Differentiation of MSCs into adipocytes and osteocytes. After applying lineage specific induction media, MSCs (A) differentiated into adipocytes, demonstrated by presence of Oil-Red O lipid droplet staining (B). Differentiation into the osteogenic lineage was demonstrated by Alizarin Red staining, identifying calcification of cells (C). Scale bar (A) represents 20 µm, scale bars (B,C) represent 50 µm. (TIF) [file pone.0019808.s001.tif]

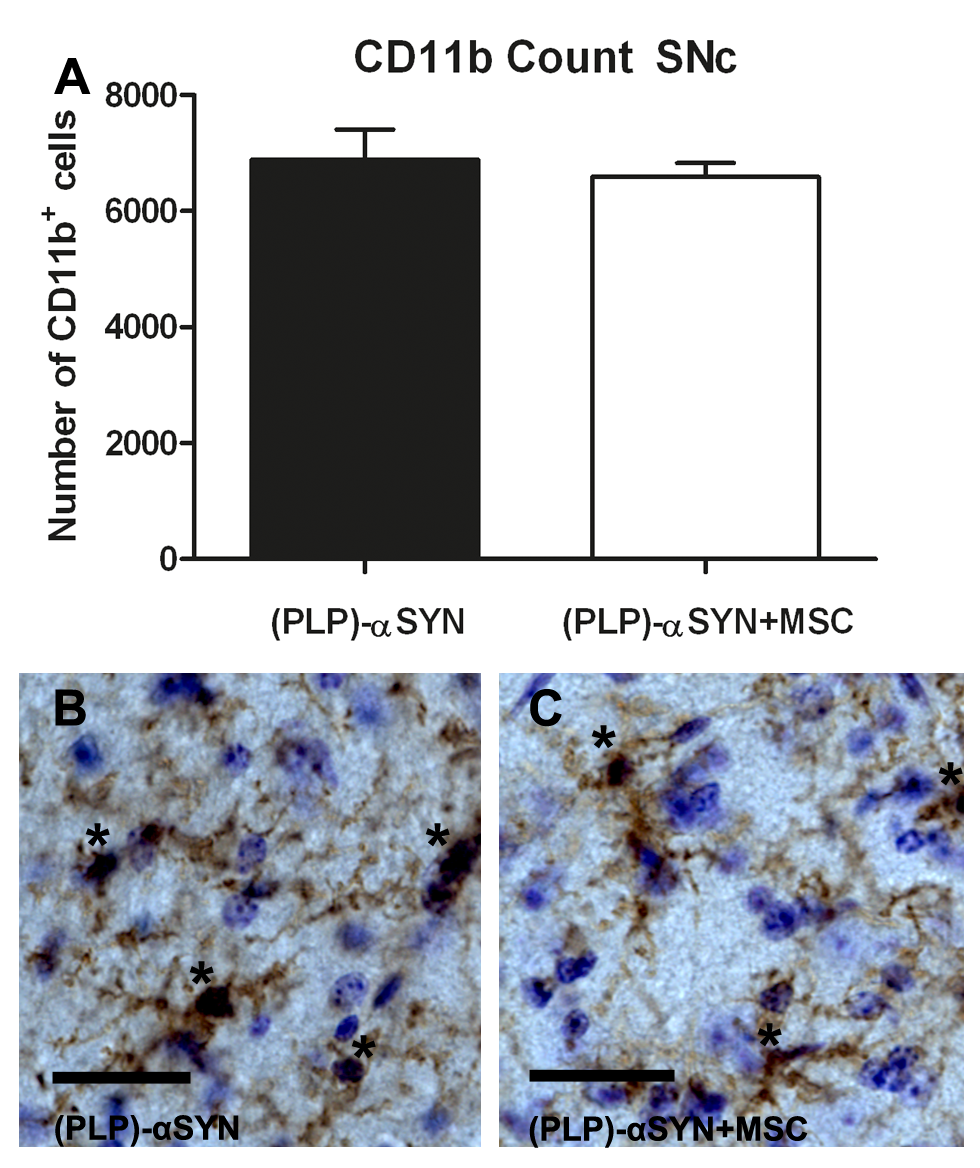

Supplement: Figure S2 — MSC treatment had no effect on the number of CD11b+ microglial cells. Quantification of CD11b+ microglial cells by stereological counting demonstrated that four weeks after MSC treatment the number of CD11b+ cells was not significantly altered in the treatment group compared to controls (p = 0.6075) (A). All data are presented as means ± SEM and were analysed by unpaired Student's t-test. Immunohistochemical staining of CD11b+ microglial cells in the SNc of a (PLP)-αSYN (n = 6) (B) and (PLP)-αSYN+MSC (n = 7) mouse (C). Scale bars (B,C) represent 20 µm. Asterisk indicate CD11b+ cells. (TIF) [file pone.0019808.s002.tif]
